# Humanin Attenuates Inflammatory Liver Injury and NF-κB Activation During Systemic Hypervirulent *Klebsiella pneumoniae* Infection

**DOI:** 10.3390/pathogens15090966

**Published:** 2026-09-10

**Authors:** Yiming Zhong, Dina Haishaer, Weidong Cai, Lin Lin, Zhaopei Guo, Yan Pan, Xiangjun Tang, Weiquan You, Ya Fu, Qishui Ou

**Affiliations:** 1 Department of Laboratory Medicine, The First Affiliated Hospital of Fujian Medical University, Fuzhou 350005, China; ymzhong@csu.edu.cn (Y.Z.);; 2 Department of Clinical Laboratory, National Clinical Research Center for Geriatric Disorders, Xiangya Hospital, Central South University, Changsha 410008, China; 3 Fujian Key Laboratory of Laboratory Medicine, The First Affiliated Hospital of Fujian Medical University, Fuzhou 350005, China; 4 Department of Laboratory Medicine, National Regional Medical Center, Binhai Campus of the First Affiliated Hospital, Fujian Medical University, Fuzhou 350200, China; 5 Department of Pathology, The First Affiliated Hospital of Fujian Medical University, Fuzhou 350005, China; caiweidong529@163.com

**Keywords:** Humanin, hypervirulent *Klebsiella pneumoniae*, bloodstream infection, inflammatory liver injury, macrophage-associated inflammation, NF-κB signaling

## Abstract

Hypervirulent *Klebsiella pneumoniae* (hvKP) causes invasive infections, including bloodstream infections and liver abscesses, and dysregulated host inflammation may exacerbate liver injury. We investigated whether pretreatment with Humanin, a mitochondria-derived anti-inflammatory peptide, could attenuate hvKP-associated liver injury. We measured serum Humanin in 20 patients with hvKP-associated bloodstream infection and liver abscess and 20 matched healthy controls. C57BL/6 mice received intraperitoneal Humanin (2.5 or 5 mg/kg/day) for five days before intravenous hvKP challenge. We assessed liver injury, inflammatory responses, hepatic transcriptomes, NF-κB activation, and survival. Serum Humanin was higher in patients than in the controls (*p* < 0.001). In infected mice, Humanin pretreatment was associated with less prominent histopathological liver injury and lower serum ALT, AST, and PCT, hepatic myeloperoxidase activity, F4/80 immunoreactivity, CD86 fluorescence, and pro-inflammatory gene expression. The effects were generally greater at 5 mg/kg than at 2.5 mg/kg. Transcriptomic analysis showed attenuation of inflammatory pathways, including NF-κB signaling (NES = −1.86, *p* = 0.002, FDR = 0.012). Humanin also reduced p-p65/p65 and p-IκBα/IκBα ratios in liver tissue and hvKP-stimulated bone-marrow-derived macrophages; 5 mg/kg prolonged survival (*p* < 0.05). Humanin pretreatment may therefore limit inflammatory liver injury during systemic hvKP infection. This effect was associated with reduced macrophage-associated inflammation and NF-κB activation, although its molecular targets remain unknown.

## 1. Introduction

Hypervirulent *Klebsiella pneumoniae* (hvKP) causes severe invasive infections, including bloodstream infection (BSI) and liver abscesses [1,2,3,4]. Compared with classical *K. pneumoniae*, hvKP causes more extensive tissue dissemination and is associated with substantial morbidity and mortality [4,5]. The convergence of hypervirulence and antimicrobial resistance, particularly in carbapenem-resistant hvKP, complicates clinical management [6,7,8,9]. Antimicrobial therapy is essential for pathogen control, but organ injury during hvKP infection may also result from dysregulated host inflammation. The liver is a major target during invasive hvKP infection, and excessive inflammation can worsen tissue damage. Adjunctive strategies that limit hepatic inflammation could therefore complement antimicrobial therapy and reduce hvKP-associated liver injury [10].

Macrophages are important components of host defense against hvKP and contribute to pathogen clearance [11,12]. However, how macrophages contribute to hvKP-induced liver injury and abscess formation remains unclear. Cao et al. found that inhibiting NF-κB activation prevented the formation of liver abscesses in an hvKP model, linking excessive innate immune activation to hepatic pathology [13].

Humanin is a mitochondria-derived peptide with established cytoprotective, anti-apoptotic, anti-inflammatory, and antioxidative properties [14,15]. Recent evidence also suggests that Humanin contributes to inflammation resolution, while its derivative S14G-Humanin reduces macrophage-derived pro-inflammatory cytokine production in mono-sodium urate crystal-induced gouty arthritis [15,16]. Whether Humanin regulates host inflammation during hvKP infection, particularly hepatic immunopathology, remains unknown. We hypothesized that Humanin pretreatment would attenuate inflammatory liver injury during systemic hvKP infection and be associated with reduced pro-inflammatory macrophage responses and NF-κB activation. We first measured serum Humanin concentrations in patients with hvKP-associated bloodstream infection and liver abscess and then tested exogenous Humanin pretreatment in an intravenous mouse infection model. We assessed liver pathology, macrophage-associated markers, inflammatory mediators, hepatic transcriptomic profiles, NF-κB activation, and survival to determine whether Humanin limits inflammatory liver injury in this setting.

## 2. Materials and Methods

### 2.1. Study Participants

This study analyzed residual blood specimens from 40 individuals collected at the First Affiliated Hospital of Fujian Medical University, including 20 patients with hvKP-associated bloodstream infection and liver abscess and 20 age- and sex-matched healthy controls undergoing routine health checkups. All samples were residual specimens that remained following routine clinical laboratory testing or routine health examinations, and all samples were de-identified before being used in this research. No additional blood collection, intervention, or procedure was performed specifically for this study. Relevant clinical information, including disease severity, comorbidities, organ dysfunction, antimicrobial treatment, and timing of serum sampling relative to diagnosis and treatment, was obtained from the participants’ medical records; this information is summarized in Supplementary Table S1. This study was performed in line with the principles of the 1975 Declaration of Helsinki, and it was approved by the Ethics Committee of the First Affiliated Hospital of Fujian Medical University (approval No. ECFAH of FMU [2021] 041).

### 2.2. Measurement of Serum Humanin Using ELISA

Serum Humanin concentrations were measured using a Humanin enzyme-linked immunosorbent assay (ELISA) kit (CUSABIO, Cat. No. CSB-EL015084HU, Wuhan, China), according to a previously described protocol [17]. According to the manufacturer, the assay had a detection range of 28–1800 pg/mL, a sensitivity of 7 pg/mL, an intra-assay coefficient of variation (CV) of <8%, and an inter-assay CV of <10%. Serum samples were diluted 1:5 with the supplied Sample Diluent before analysis. Briefly, standards and serum samples were added to antibody-precoated wells and incubated at 37 °C, followed sequentially by biotin-conjugated anti-Humanin antibody and horseradish peroxidase (HRP)-conjugated avidin with washing between the indicated steps. TMB substrate was then added for color development; the reaction was stopped with stop solution, and optical density (OD) was measured at 450 nm using a Multiskan SkyHigh microplate reader (Thermo Fisher Scientific, Waltham, MA, USA). Humanin concentrations were calculated from the standard curve and corrected for the dilution factor.

### 2.3. Bacterial Culture

The clinical *Klebsiella pneumoniae* isolate used in this study was classified as hvKP, based specifically on the presence of five virulence genes previously reported to distinguish hvKP from classical *K. pneumoniae*: *iucA*, *iroB*, *peg-344*, *rmpA*, and *rmpA2* [18]. The isolate was cultured in Luria–Bertani (LB) broth (Solarbio, Beijing, China) at 37 °C and shaken at 180 rpm [19]. Bacterial density was estimated via the measurement of the optical density at 600 nm. Late-log-phase bacteria were used for the mouse and cell infection experiments.

### 2.4. Mouse Model of Systemic hvKP Infection and Humanin Pretreatment

Eight-week-old male C57BL/6 mice were used in the experiments; group sizes are reported in the figure legends. Mice were purchased from Vital River Laboratory Animal Technologies Co., Ltd. (Foshan, China). Sample sizes were selected based on those used in previous similar experiments and our prior experimental experience; no formal a priori power calculation was performed. For each experiment, the mice were randomly allocated to experimental groups using computer-generated random numbers. The mice were housed under specific pathogen-free conditions in a temperature-controlled room with appropriate ventilation. All animal procedures were conducted in accordance with the applicable Chinese regulations and institutional guidelines for the care and use of laboratory animals and approved by the Ethics Committee of the First Affiliated Hospital of Fujian Medical University (approval No. ECFAH of FMU [2021] 041).

The mice received Humanin intraperitoneally at 2.5 or 5 mg/kg once daily for five consecutive days. Then, they were challenged through the tail vein with 3 × 10^2^ colony-forming units (CFU) of hvKP. Twenty-four hours after being infected, mice were sacrificed via CO_2_ inhalation. To determine the bacterial burden in vivo, blood and liver tissues were collected aseptically. A standardized portion of each liver tissue sample was weighed, homogenized in sterile PBS, and serially diluted. Blood samples were also serially diluted in PBS. The diluted blood samples and liver homogenates were then plated onto Mueller–Hinton agar (MHA) plates (Thermo Fisher Scientific, Waltham, MA, USA). Following overnight incubation at 37 °C, bacterial colonies were enumerated, and bacterial burdens were expressed as log10 CFU/mL of blood and log10 CFU/g of liver tissue. The remaining liver tissues were allocated for histological, immunological, and molecular analyses. Tissues intended for histological examination were fixed, whereas tissues for molecular analysis were rapidly frozen in liquid nitrogen-cooled isopentane and stored at −80 °C until further analysis. Humanin (sequence: MAPRGFSCLLLLTSEIDLPVKRRA; purity > 98%) was synthesized by China Peptides Co., Ltd. (Suzhou, China).

For the survival experiment, mice received the same Humanin pretreatment and hvKP challenge, though these mice were monitored for 96 h. The 24 h tissue-collection experiment and the survival experiment were each conducted as a single experimental batch using separate cohorts of mice.

### 2.5. Immunohistochemistry and Histology

Mouse livers were fixed in 4% paraformaldehyde and embedded in paraffin. Sections were dewaxed and subjected to heat-induced epitope retrieval, as previously described [20]. After blocking, sections were incubated overnight at 4 °C with an F4/80 (D2S9R) rabbit monoclonal antibody (1:150, #70076, Cell Signaling Technology, Danvers, MA, USA), followed by a goat anti-rabbit secondary antibody. Immunoreactivity was visualized with DAB, and nuclei were counterstained with hematoxylin. For the histological assessment, liver sections were stained using hematoxylin and eosin (H&E) and evaluated qualitatively for inflammatory cell infiltration and hepatocellular necrosis. No standardized quantitative histopathological scoring system was applied. The F4/80-positive area was quantified in ImageJ (version 1.54p).

### 2.6. Immunofluorescence Staining

Immunofluorescence staining was performed as previously described [21]. Paraffin-embedded liver sections were dewaxed and subjected to antigen retrieval. After blocking, sections were incubated overnight at 4 °C with CD86 (E5W6H) rabbit monoclonal antibody (1:400, #19589, Cell Signaling Technology, Danvers, MA, USA) or CD206 (E6T5J) rabbit monoclonal antibody (1:400, #24595, Cell Signaling Technology, Danvers, MA, USA). After thorough washing, sections were incubated with a Cy3-conjugated secondary antibody (Abcam, Cambridge, UK). Nuclei were counterstained with DAPI. Sections were observed and imaged using a fluorescence scanner (G cell technology, Guangzhou, China). Histological assessment and quantitative analyses of immunohistochemical and immunofluorescence images were performed by an investigator who was unaware of the treatment allocation.

### 2.7. Clinical Biochemistry

Serum alanine aminotransferase (ALT) and aspartate aminotransferase (AST) were measured using an ADVIA 2400 automated biochemical analyzer (Siemens, Erlangen, Germany).

### 2.8. Measurement of PCT

Mouse serum procalcitonin (PCT) was measured using an ELISA according to the manufacturer’s instructions (Elabscience, #E-EL-M2419, Wuhan, China).

### 2.9. Myeloperoxidase (MPO) Assay

Hepatic myeloperoxidase (MPO) activity was used as an index of inflammatory cell infiltration. Liver tissue was homogenized in buffer at a 1:19 weight-to-volume ratio, and MPO activity was measured using a detection kit (ZCIBIO, ZC-S0923, Shanghai, China). Absorbance was recorded at 460 nm.

### 2.10. BMDM Culture and hvKP Stimulation

Bone marrow cells were isolated from the femurs and tibias of healthy C57BL/6 mice and cultured at 37 °C with 5% CO_2_. Each biological replicate in the BMDM experiments was generated from bone marrow cells derived from an independent mouse. The culture medium used was Dulbecco’s Modified Eagle Medium (DMEM, MeilunBio, Dalian, China) supplemented with 10% fetal bovine serum, 1% penicillin/streptomycin, and 40 ng/mL macrophage colony-stimulating factor (CB34, Novoprotein, Suzhou, China). Bone-marrow-derived macrophages (BMDMs) were collected on day 7. Cells were pretreated for 2 h with 25 or 50 μM Humanin dissolved in Dulbecco’s phosphate-buffered saline (DPBS) and then stimulated with hvKP at a multiplicity of infection of 10 for 30 min. The Humanin concentrations were selected based on a previous in vitro study demonstrating anti-inflammatory effects at 25 and 50 μM [22].

### 2.11. Protein Extraction and Western Blotting

Tissues were disrupted using a cryogenic grinder, and cells were lysed on ice for 30 min in RIPA buffer (Solarbio, Beijing, China) containing protease and phosphatase inhibitors (BOSTER, Wuhan, China). Lysates were centrifuged at 12,000× *g* for 30 min at 4 °C. Protein concentrations were determined using a BCA kit (Beyotime, Shanghai, China). For each sample, 50 μg of protein was separated by SDS–polyacrylamide gel electrophoresis and transferred to polyvinylidene difluoride membranes (Bio-Rad, Hercules, CA, USA). After transfer, the membranes were blocked with 5% non-fat milk and incubated overnight at 4 °C with primary antibodies. The primary antibodies used in this study were phospho-NF-κB p65 (Ser536) rabbit monoclonal antibody (1:1000, #3033, Cell Signaling Technology, Danvers, MA, USA), NF-κB p65 (D14E12) rabbit monoclonal antibody (1:1000, #8242, Cell Signaling Technology, Danvers, MA, USA), phospho-IκBα (Ser32) rabbit monoclonal antibody (1:1000, #2859, Cell Signaling Technology, Danvers, MA, USA), and IκBα (L35A5) mouse monoclonal antibody (1:1000, #4814, Cell Signaling Technology, Danvers, MA, USA). GAPDH (14C10) rabbit monoclonal antibody (1:1000, #2118, Cell Signaling Technology, Danvers, MA, USA) served as the loading control. Following incubation with primary antibodies, the membranes were incubated with horseradish peroxidase (HRP)-conjugated secondary antibodies (1:8000, Beyotime, Shanghai, China). Signals were visualized using an enhanced chemiluminescence (ECL) immunoblotting detection system (Biosharp, Beijing, China). After the detection of phosphorylated proteins, the membranes were stripped and reprobed with antibodies against the corresponding total proteins. Band intensities were quantified in ImageJ. Phosphorylation was expressed as the p-p65/total p65 and p-IκBα/total IκBα ratios.

### 2.12. RNA Extraction and Quantitative Real-Time PCR

Total RNA was extracted from liver tissue using an RNA extraction kit (Accurate Biology, Changsha, China), and its concentration was measured with a NanoDrop spectrophotometer (Thermo Fisher Scientific, Waltham, MA, USA). One microgram of RNA was reverse-transcribed using a cDNA synthesis kit (Vazyme, Nanjing, China). Quantitative real-time PCR was performed on a QuantStudio 5 96-well Real-Time PCR System (Applied Biosystems, Thermo Fisher Scientific, Waltham, MA, USA). *Gapdh* served as the reference gene, and relative expression was calculated using the 2^−ΔΔCt^ method. Primer sequences are listed in Table 1.

### 2.13. RNA Sequencing

Majorbio Biotechnology Company (Majorbio, Shanghai, China) extracted RNA from liver tissue, assessed RNA quality, prepared sequencing libraries, and performed transcriptome sequencing. After quality filtering, each library yielded 40.09–50.51 million clean reads, corresponding to 6.00–7.54 Gb of clean sequence data, with Q30 values ranging from 97.76% to 97.95%. Clean reads were aligned to the *Mus musculus* GRCm39 reference genome, with mapping rates ranging from 96.55% to 97.29%. Differentially expressed genes were identified with DESeq2 (version 1.42.0) using thresholds of |log2 fold change| ≥ 1 and adjusted *p* < 0.05. Gene Ontology and Kyoto Encyclopedia of Genes and Genomes enrichment analyses and gene-set enrichment analysis were performed on the Majorbio Cloud Platform (https://www.majorbio.com/).

### 2.14. Statistical Analysis

Statistical analyses were performed in GraphPad Prism 10.0 or SPSS 25. Normality was assessed using the Shapiro–Wilk test. Non-normally distributed data from the two-group comparisons were analyzed using the two-tailed Mann–Whitney U test. For multiple-group comparisons, homogeneity of variance was evaluated using the Brown–Forsythe test. Data satisfying the assumptions for ordinary one-way ANOVA were analyzed using one-way ANOVA followed by Tukey’s multiple-comparisons test. When the homogeneity-of-variance assumption was not satisfied, Welch’s one-way ANOVA followed by Dunnett’s T3 multiple-comparisons test was used. Survival distributions were compared with the log-rank test. Overall effect sizes for ordinary one-way ANOVA were expressed as eta squared (η^2^), and mean differences with 95% confidence intervals were provided for the corresponding pairwise comparisons. *p* < 0.05 was considered statistically significant.

## 3. Results

### 3.1. Serum Humanin Concentrations Are Higher in Patients with hvKP-Associated Bloodstream Infection and Liver Abscess

**Figure 1:**
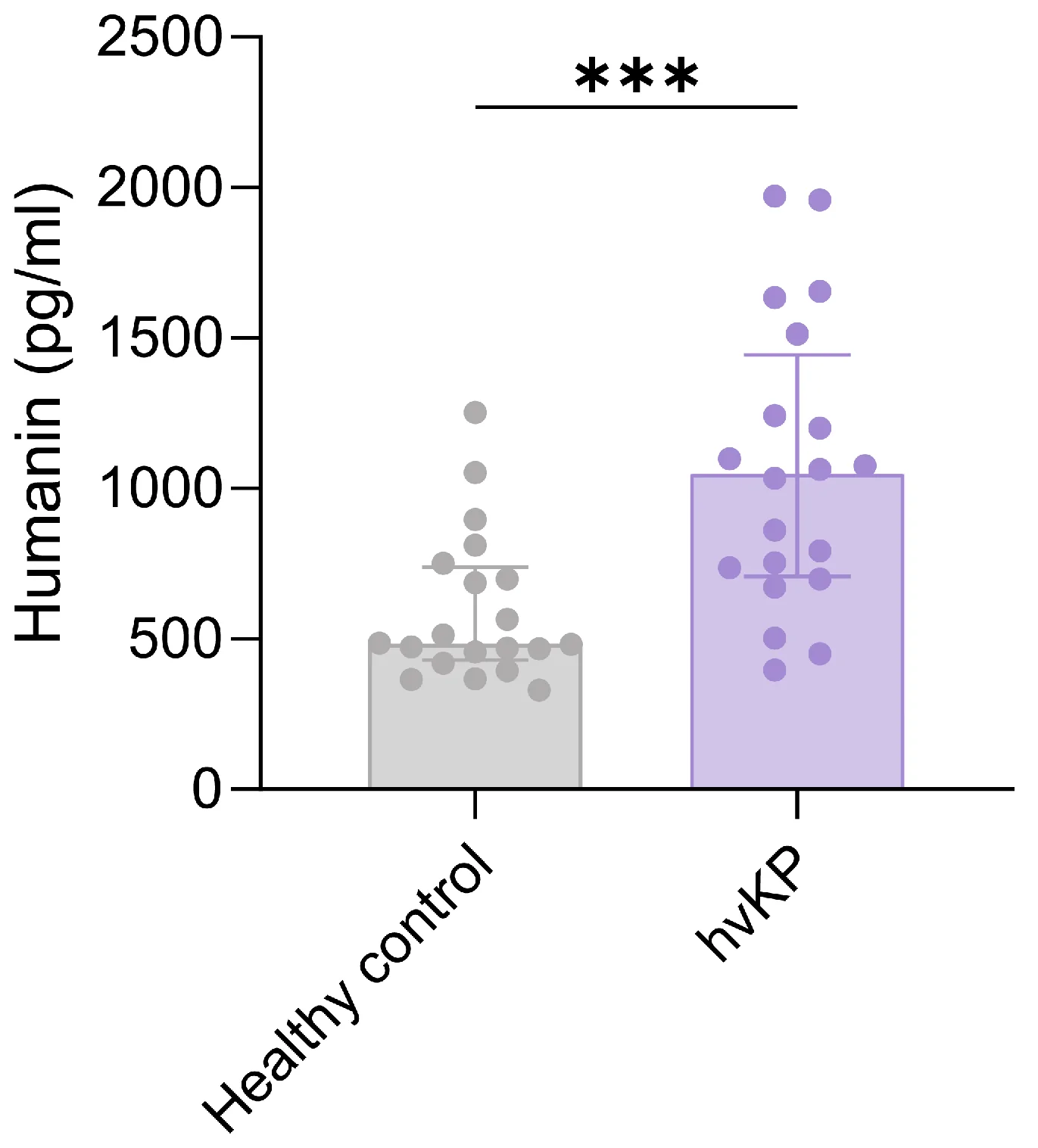
Serum Humanin concentrations in patients with hvKP-associated bloodstream infection and liver abscess compared with healthy controls. Each point represents one participant, and data are presented as the median with interquartile range (IQR) (*n* = 20 per group). Statistical comparison was performed using a two-tailed Mann–Whitney U test. ***, *p* < 0.001; hvKP, hypervirulent *Klebsiella pneumoniae*.

The clinical characteristics of the patients are summarized in Supplementary Table S1. We compared circulating Humanin concentrations between patients with hvKP-associated bloodstream infection and liver abscess and healthy controls. The serum Humanin concentrations were significantly higher in the patient group than in the healthy controls (median [IQR], 1049.0 [727.5–1310.3] vs. 484.4 [448.7–712.8] pg/mL; Hodges–Lehmann location shift, 388.9 pg/mL; 95% CI, 202.4–686.9 pg/mL; *p* < 0.001; Figure 1).

### 3.2. Humanin Attenuates Liver Injury During Systemic hvKP Infection in Mice

**Figure 2:**
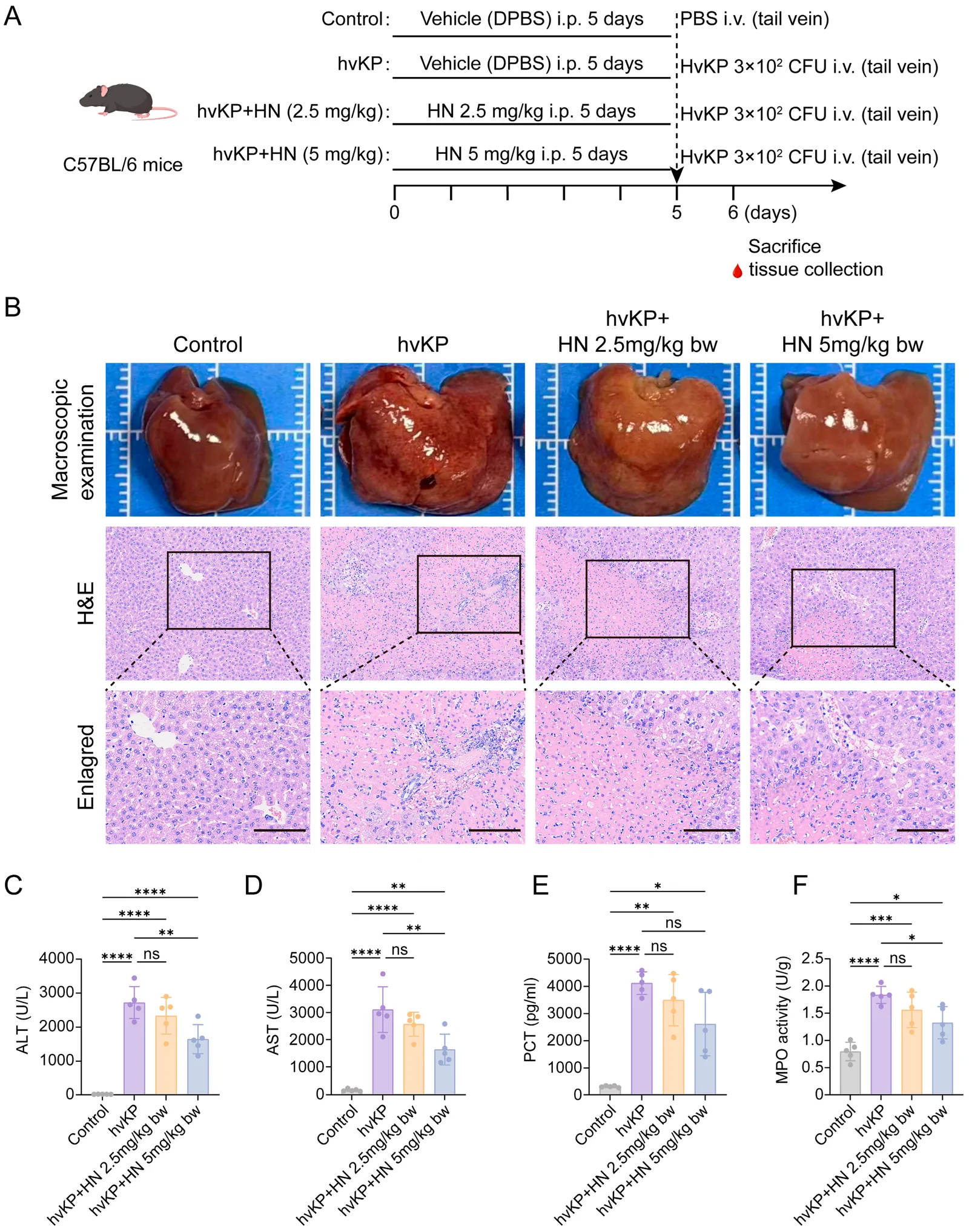
Humanin attenuates liver injury and inflammatory responses during systemic hvKP infection in mice. (**A**) Schematic of the experimental design. C57BL/6 mice received daily intraperitoneal injections of Humanin at 2.5 or 5 mg/kg body weight, or vehicle (DPBS), for five consecutive days. Mice were subsequently challenged intravenously through the tail vein with 3 × 10^2^ CFU of hvKP; uninfected control mice received PBS. Samples were collected at 24 h after infection. (**B**) Representative gross images and H&E-stained sections of mouse livers. Boxed regions are shown at higher magnification in the lower panels. Scale bars, 100 μm. (**C**–**F**) Serum ALT (**C**), AST (**D**), and PCT concentrations (**E**), and hepatic MPO activity (**F**). Each point represents one mouse, and bars show the mean ± SD (*n* = 5 per group). Statistical comparisons in panels (**C**,**D**,**F**) were performed using ordinary one-way ANOVA followed by Tukey’s multiple-comparisons test, whereas panel E was analyzed using Welch’s one-way ANOVA followed by Dunnett’s T3 multiple-comparisons test. *, *p* < 0.05; **, *p* < 0.01; ***, *p* < 0.001; ****, *p* < 0.0001; ns, not significant. HN, Humanin; hvKP, hypervirulent *Klebsiella pneumoniae*; CFU, colony-forming units; i.p., intraperitoneal; i.v., intravenous; bw, body weight.

Mice were pretreated with Humanin at 2.5 or 5 mg/kg for five consecutive days before intravenous challenge with 3 × 10^2^ CFU of hvKP (Figure 2A). Twenty-four hours after infection, untreated hvKP-infected mice showed inflammatory cell infiltration and focal hepatic necrosis. Qualitative examination of H&E-stained liver sections showed that these histopathological changes were less prominent in Humanin-pretreated mice, particularly at 5 mg/kg (Figure 2B).

Compared with uninfected controls, hvKP-infected mice showed marked increases in serum ALT and AST levels, consistent with hepatocellular injury (Figure 2C,D). hvKP infection also increased serum PCT levels and hepatic MPO activity, indicating an enhanced systemic inflammatory response and hepatic inflammatory cell infiltration (Figure 2E,F). Humanin pretreatment was associated with lower ALT, AST, PCT, and hepatic MPO values, with generally larger reductions at 5 mg/kg. At 5 mg/kg, the reductions in ALT, AST, and hepatic MPO activity were statistically significant compared with the untreated hvKP group, whereas the reduction in PCT did not reach statistical significance (Figure 2C–F). The overall effect sizes and mean differences with 95% confidence intervals for the corresponding group comparisons are provided in Supplementary Table S2. No statistically significant differences in blood or hepatic bacterial burden were detected between the Humanin-pretreated and untreated hvKP groups (Supplementary Figure S1 and Supplementary Table S2).

### 3.3. Humanin Attenuates Hepatic Macrophage-Associated Inflammation During Systemic hvKP Infection

**Figure 3:**
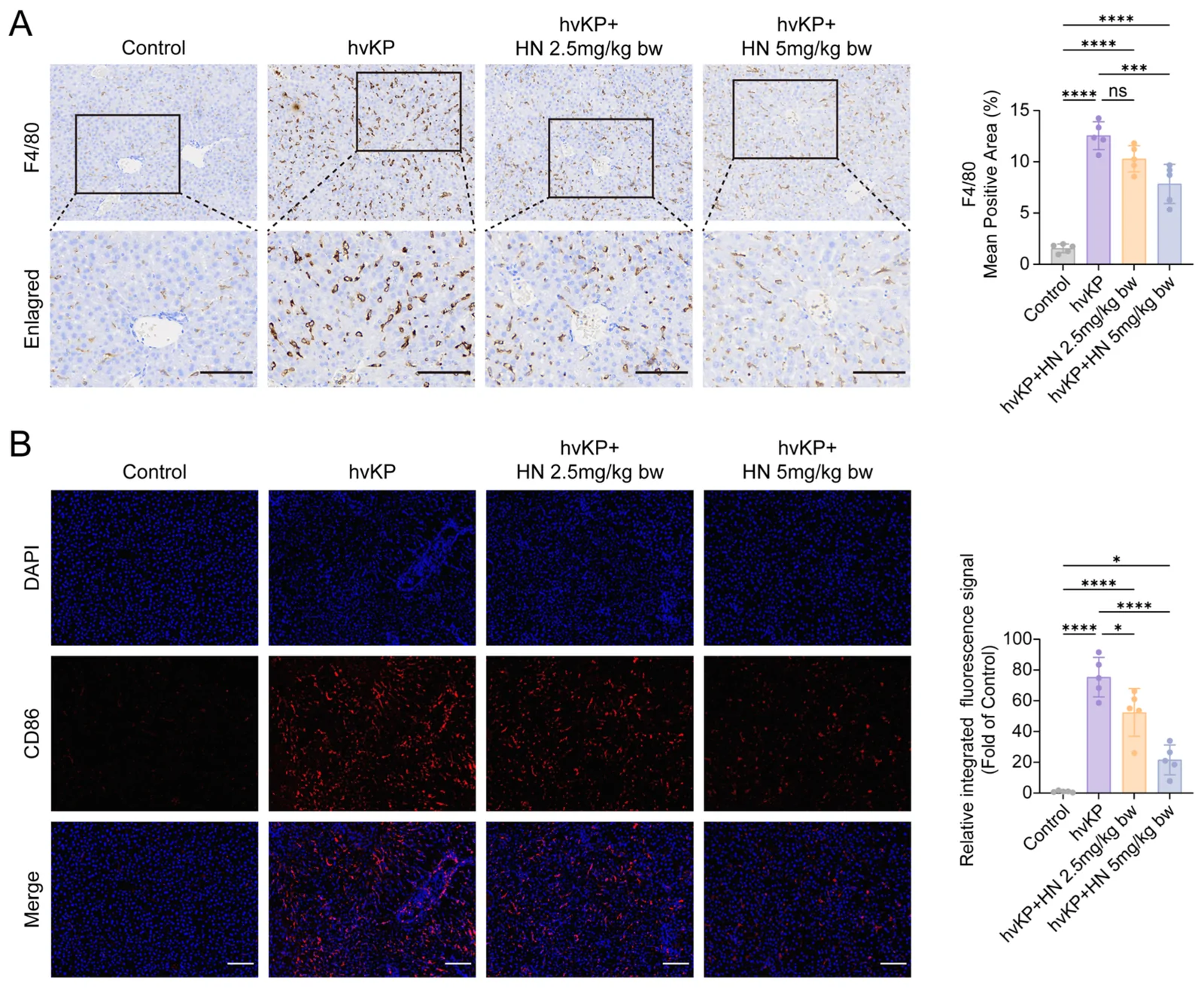
Humanin reduces hepatic F4/80 immunoreactivity and CD86 expression during systemic hvKP infection. (**A**) Representative immunohistochemical images of F4/80 staining in liver sections from the indicated groups and quantification of the F4/80-positive area. Boxed regions are shown at higher magnification in the lower panels. (**B**) Representative immunofluorescence images of hepatic CD86 staining and quantification of the integrated CD86 fluorescence intensity relative to the uninfected control group. CD86 is shown in red, nuclei were counterstained with DAPI (blue), and merged images are shown in the lower row. Scale bars, 100 μm. Each point represents one mouse, and bars show the mean ± SD (*n* = 5 per group). Statistical comparisons in panels A and B were performed using ordinary one-way ANOVA followed by Tukey’s multiple-comparisons test. *, *p* < 0.05; ***, *p* < 0.001; ****, *p* < 0.0001; ns, not significant. HN, Humanin; hvKP, hypervirulent *Klebsiella pneumoniae*; bw, body weight.

We assessed hepatic macrophage-associated inflammatory responses via immunohistochemical staining for F4/80 and immunofluorescence staining for CD86 and CD206. Compared with the uninfected controls, hvKP-infected mice showed a marked increase in the hepatic F4/80-positive area (Figure 3A). Humanin pretreatment reduced F4/80 immunoreactivity in a dose-related pattern. The reduction was modest and did not reach statistical significance at 2.5 mg/kg, whereas a significant reduction was observed at 5 mg/kg.

hvKP infection also markedly increased hepatic CD86 fluorescence intensity (Figure 3B). Humanin pretreatment reduced the CD86 signal, with a greater reduction observed at 5 mg/kg. In contrast, CD206 fluorescence intensity did not differ significantly among the four groups (Supplementary Figure S2). Overall effect sizes and mean differences with 95% confidence intervals for the corresponding group comparisons are provided in Supplementary Table S2.

### 3.4. Humanin Suppresses Hepatic Pro-Inflammatory Gene Expression During Systemic hvKP Infection

**Figure 4:**
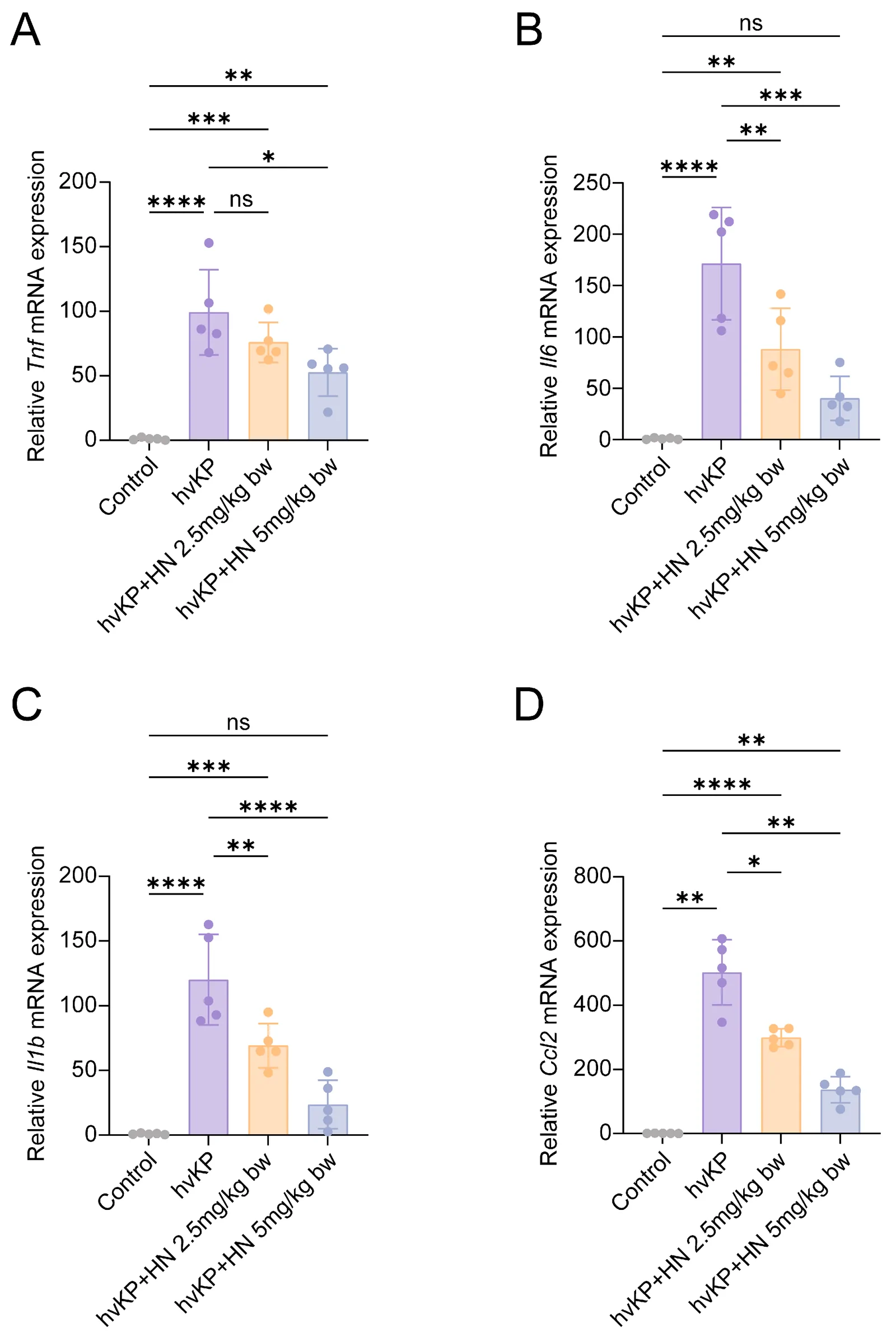
Humanin reduces hepatic pro-inflammatory gene expression during systemic hvKP infection. Relative hepatic mRNA expression of *Tnf* (**A**), *Il6* (**B**), *Il1b* (**C**), and *Ccl2* (**D**) was measured by quantitative real-time PCR. Gene expression was normalized to *Gapdh* and calculated using the 2^−ΔΔCt^ method relative to the uninfected control group. Each point represents one mouse, and bars show the mean ± SD (*n* = 5 per group). Statistical comparisons in panels (**A**–**C**) were performed using ordinary one-way ANOVA followed by Tukey’s multiple-comparisons test, whereas panel (**D**) was analyzed using Welch’s one-way ANOVA followed by Dunnett’s T3 multiple-comparisons test. *, *p* < 0.05; **, *p* < 0.01; ***, *p* < 0.001; ****, *p* < 0.0001; ns, not significant. HN, Humanin; hvKP, hypervirulent *Klebsiella pneumoniae*; bw, body weight.

We then measured hepatic *Tnf*, *Il6*, *Il1b*, and *Ccl2* mRNA using quantitative real-time PCR. Compared with uninfected controls, hvKP-infected mice showed marked increases in the expression of all four genes (Figure 4A–D). Humanin pretreatment at 2.5 mg/kg significantly reduced *Il6*, *Il1b*, and *Ccl2* expression, whereas the reduction in *Tnf* expression did not reach statistical significance. At 5 mg/kg, Humanin significantly reduced the expression of all four pro-inflammatory genes compared with untreated hvKP-infected mice. The reductions were generally greater at 5 mg/kg than at 2.5 mg/kg, indicating a dose-related pattern of suppression (Figure 4A–D). The overall effect sizes and mean differences with 95% confidence intervals for the corresponding group comparisons are provided in Supplementary Table S2.

### 3.5. Humanin Suppresses Hepatic Inflammatory Transcriptional Programs During Systemic hvKP Infection

**Figure 5:**
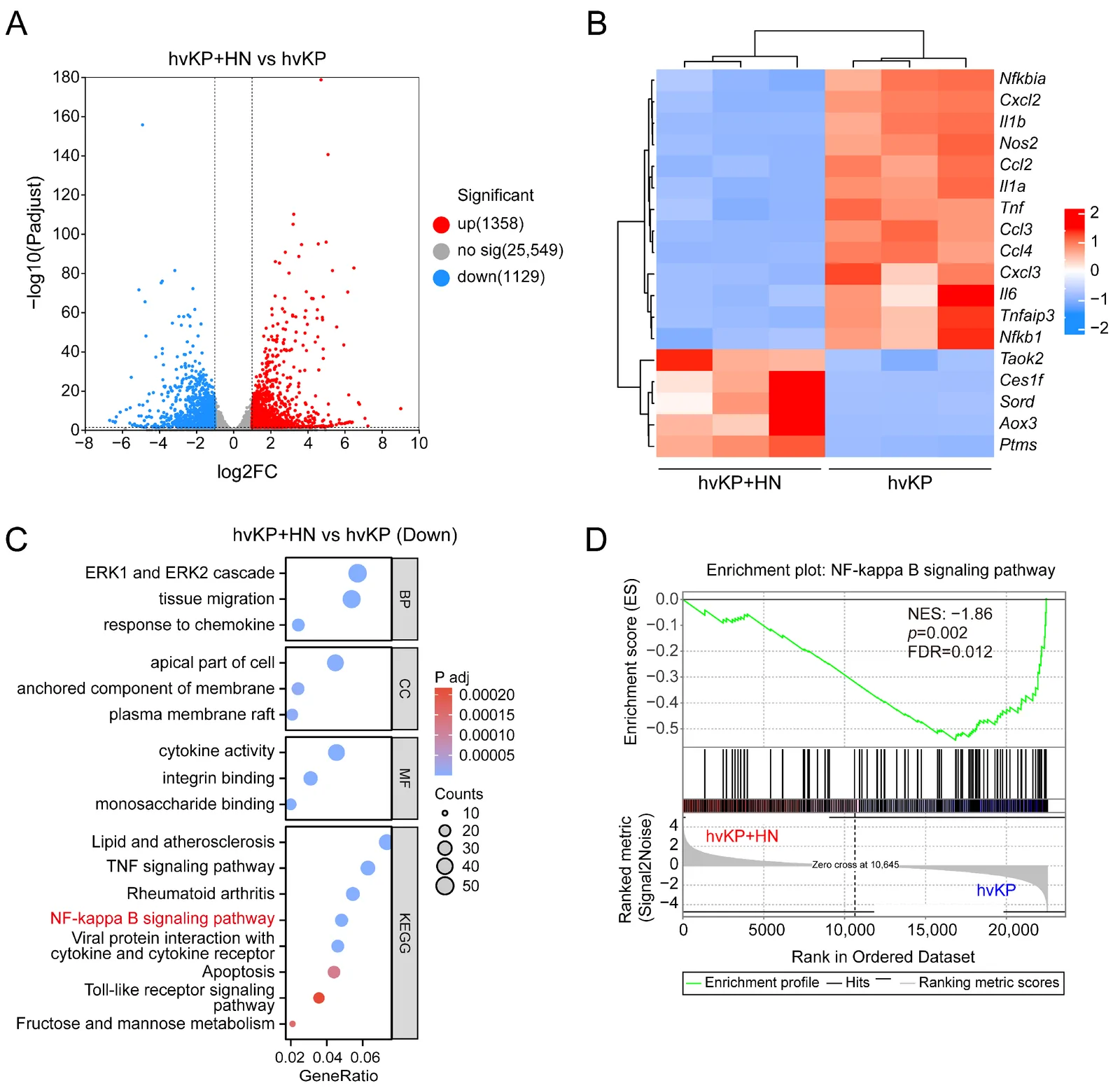
Humanin attenuates hepatic inflammatory transcriptional programs during systemic hvKP infection. Liver RNA sequencing was performed to compare untreated hvKP-infected mice with hvKP-infected mice pretreated with Humanin at 5 mg/kg. (**A**) Volcano plot showing differentially expressed genes in the Humanin-pretreated group relative to the hvKP group. Red points indicate 1358 upregulated genes, blue points indicate 1129 downregulated genes, and gray points indicate 25,549 genes that did not meet the differential-expression criteria. Differentially expressed genes were identified using thresholds of |log_2_ fold change| ≥ 1 and adjusted *p* < 0.05. (**B**) Hierarchical clustering heatmap of selected inflammation-associated differentially expressed genes. (**C**) Gene Ontology and Kyoto Encyclopedia of Genes and Genomes enrichment analyses of genes downregulated in the Humanin-pretreated group relative to the hvKP group. Dot size represents the number of enriched genes, the *x*-axis indicates the gene ratio, and color indicates the adjusted *p*-value. (**D**) Gene-set enrichment analysis showing negative enrichment of the NF-κB signaling pathway in the Humanin-pretreated group relative to the hvKP group (NES = −1.86, *p* = 0.002, FDR = 0.012). The dashed vertical line in panel D indicates the zero-crossing point of the ranked metric. no sig, not significant; HN, Humanin; hvKP, hypervirulent *Klebsiella pneumoniae*; DEG, differentially expressed gene; GO, Gene Ontology; BP, biological process; CC, cellular component; MF, molecular function; KEGG, Kyoto Encyclopedia of Genes and Genomes; GSEA, gene-set enrichment analysis; NES, normalized enrichment score; FDR, false discovery rate.

We used liver RNA sequencing to compare untreated hvKP-infected mice with hvKP-infected mice pretreated with 5 mg/kg Humanin. Principal component analysis showed that biological replicates clustered according to experimental group, with PC1 and PC2 explaining 62.14% and 16.83% of the total variance, respectively (Supplementary Figure S3). Compared with the hvKP group, the Humanin-pretreated group exhibited 2487 differentially expressed genes, of which 1358 were upregulated and 1129 were downregulated according to the prespecified differential-expression criteria (Figure 5A). Hierarchical clustering showed lower expression of multiple inflammation-associated genes in the Humanin-pretreated group, including *Il1b*, *Il6*, *Tnf*, *Ccl2*, and *Nos2* (Figure 5B).

Downregulated genes were enriched for chemokine responses, cytokine activity, TNF signaling, Toll-like receptor signaling, and NF-κB signaling (Figure 5C). Gene-set enrichment analysis likewise showed negative enrichment of NF-κB signaling in the Humanin-pretreated group relative to the hvKP group (NES = −1.86, *p* = 0.002, FDR = 0.012; Figure 5D). Humanin pretreatment was associated with broad attenuation of hepatic inflammatory transcriptional programs during systemic hvKP infection.

### 3.6. Humanin Pretreatment Attenuates hvKP-Induced NF-κB Activation in Liver Tissue and BMDMs

**Figure 6:**
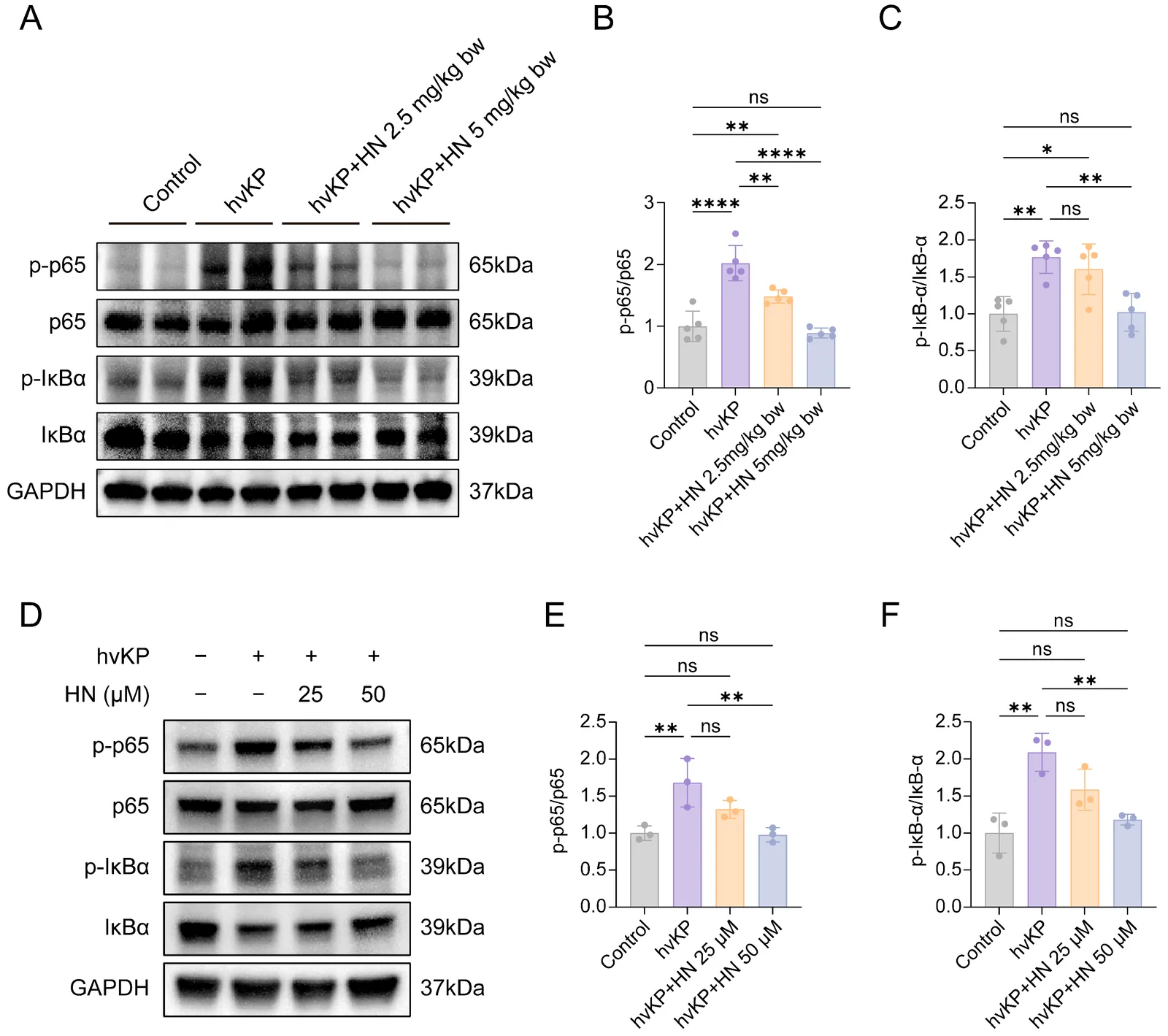
Humanin attenuates hvKP-induced NF-κB pathway activation in mouse liver tissue and bone-marrow-derived macrophages. (**A**) Representative Western blots showing p-p65, p65, p-IκBα, IκBα, and GAPDH in liver tissues from the indicated groups. (**B**,**C**) Densitometric quantification of the p-p65/p65 (**B**) and p-IκBα/IκBα (**C**) ratios in mouse liver tissue. Each point represents one mouse, and bars show the mean ± SD (*n* = 5 per group). (**D**) Representative Western blots showing p-p65, p65, p-IκBα, IκBα, and GAPDH in bone-marrow-derived macrophages (BMDMs). BMDMs were pretreated with Humanin at 25 or 50 μM for 2 h and subsequently stimulated with hvKP at a multiplicity of infection of 10 for 30 min. (**E**,**F**) Densitometric quantification of the p-p65/p65 (**E**) and p-IκBα/IκBα (**F**) ratios in BMDMs. Each point represents one independent biological replicate, and bars show the mean ± SD (*n* = 3 per group). Statistical comparisons in panels (**B**,**C**,**E**,**F**) were performed using ordinary one-way ANOVA followed by Tukey’s multiple-comparisons test. *, *p* < 0.05; **, *p* < 0.01; ****, *p* < 0.0001; ns, not significant. HN, Humanin; hvKP, hypervirulent *Klebsiella pneumoniae*; bw, body weight.

We assessed NF-κB activation in mouse liver tissue by measuring p65 and IκBα phosphorylation using Western blotting. Compared with the uninfected controls, hvKP-infected mice showed significant increases in the p-p65/p65 and p-IκBα/IκBα ratios, consistent with the activation of NF-κB signaling (Figure 6A–C). Humanin pretreatment reduced the p-p65/p65 ratio at both 2.5 and 5 mg/kg, with a greater reduction observed at 5 mg/kg. The p-IκBα/IκBα ratio showed a modest, nonsignificant decrease at 2.5 mg/kg but was significantly reduced at 5 mg/kg (Figure 6A–C).

We next tested Humanin in hvKP-stimulated bone-marrow-derived macrophages (BMDMs). BMDMs were pretreated with Humanin at 25 or 50 μM for 2 h and subsequently stimulated with hvKP at a multiplicity of infection of 10 for 30 min. hvKP stimulation increased both the p-p65/p65 and p-IκBα/IκBα ratios. Pretreatment with 25 μM Humanin produced a downward trend in both ratios, whereas 50 μM Humanin significantly reduced the phosphorylation of p65 and IκBα relative to hvKP stimulation alone (Figure 6D–F). Total p65 and IκBα protein levels normalized to GAPDH did not differ significantly among the corresponding experimental groups in either mouse liver tissue or BMDMs (Supplementary Figure S4). Humanin pretreatment attenuated hvKP-induced NF-κB activation in both liver tissue and BMDMs. Overall effect sizes and mean differences with 95% confidence intervals for the corresponding group comparisons are provided in Supplementary Table S2.

### 3.7. Humanin Pretreatment at 5 mg/kg Prolongs Survival Following Systemic hvKP Infection

**Figure 7:**
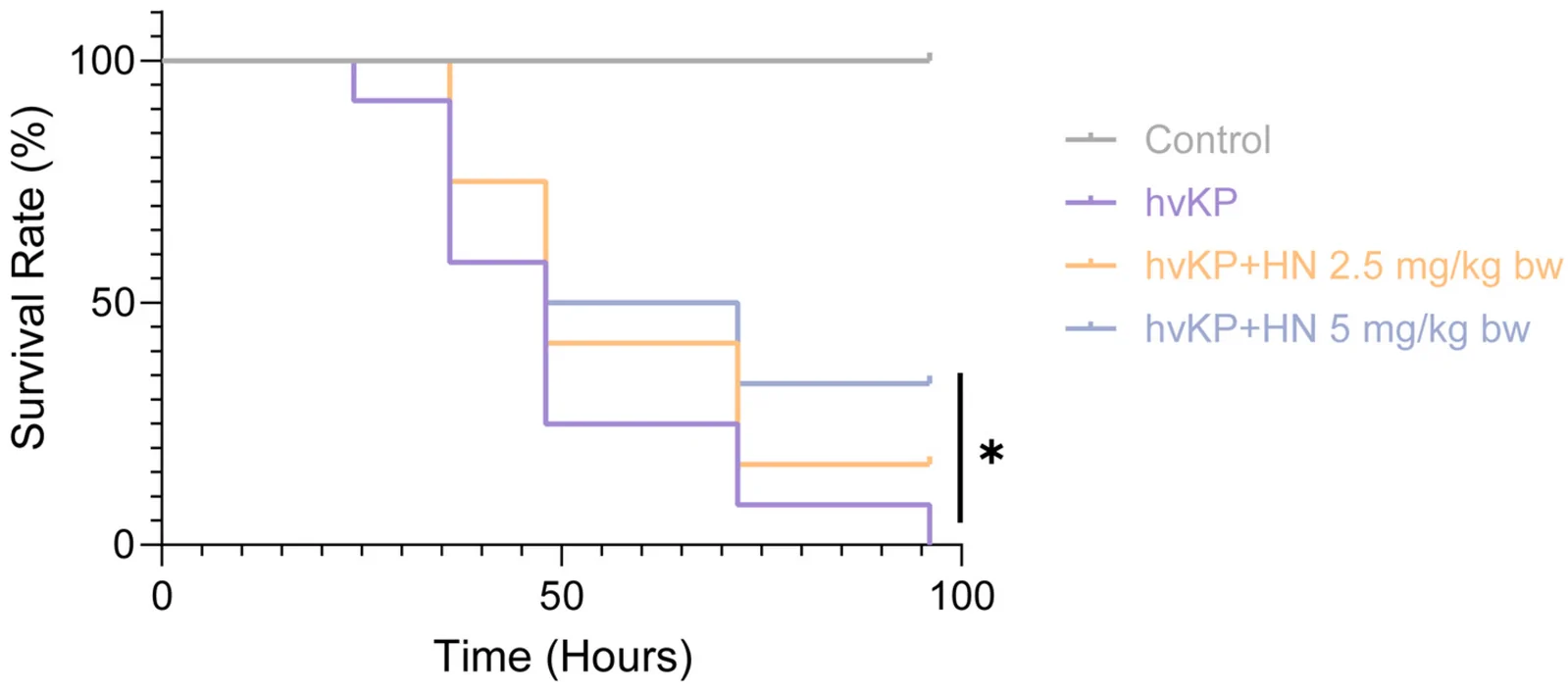
Humanin pretreatment at 5 mg/kg prolongs survival following systemic hvKP infection. Kaplan–Meier survival curves of C57BL/6 mice pretreated intraperitoneally with Humanin at 2.5 or 5 mg/kg body weight, or vehicle (DPBS), once daily for five consecutive days before intravenous challenge with 3 × 10^2^ CFU of hvKP. Uninfected control mice received PBS intravenously. Survival was monitored for 96 h after infection (*n* = 12 per group). Survival distributions were compared using the log-rank (Mantel–Cox) test. Humanin pretreatment at 5 mg/kg significantly prolonged survival compared with the hvKP group (*p* < 0.05), whereas the difference between the 2.5 mg/kg group and the hvKP group was not statistically significant. *, *p* < 0.05; HN, Humanin; hvKP, hypervirulent *Klebsiella pneumoniae*; bw, body weight.

We examined survival in a separate cohort of mice pretreated with Humanin before intravenous hvKP challenge. All uninfected control mice survived throughout the observation period, whereas all mice in the untreated hvKP group died within 96 h after infection. Humanin pretreatment at 2.5 mg/kg produced a numerical improvement in survival, but the difference relative to the hvKP group did not reach statistical significance. In contrast, pretreatment with 5 mg/kg Humanin significantly prolonged survival compared with the hvKP group (*p* < 0.05; Figure 7).

## 4. Discussion

Serum Humanin concentrations were higher in patients with hvKP-associated bloodstream infection and liver abscess. In mice, exogenous Humanin pretreatment attenuated inflammatory liver injury after systemic hvKP infection. Pretreatment improved liver histopathology, reduced biochemical and inflammatory markers, and prolonged survival at 5 mg/kg. Liver transcriptomics and reduced phosphorylation of p65 and IκBα in liver tissue and hvKP-stimulated BMDMs were consistent with attenuation of inflammatory signaling. Humanin pretreatment attenuated liver injury without a statistically significant reduction in blood or hepatic bacterial burden under the conditions tested. Although this finding does not establish equivalence between groups or prove a host-directed mechanism, the accompanying reduction in inflammatory markers is consistent with previous work implicating macrophages and excessive innate immune activation in hvKP-associated hepatic injury [13,23].

Humanin is a mitochondria-derived peptide originally identified for its anti-apoptotic and neuroprotective properties [14]. Evidence from several disease models indicates that Humanin also has cytoprotective, anti-inflammatory, and antioxidative effects. Humanin produced by efferocytic macrophages contributes to inflammation resolution, while its derivative S14G-Humanin reduces macrophage-derived pro-inflammatory cytokine production in experimental gouty arthritis [15,16]. Humanin has also been detected in human atherosclerotic plaques, and exogenous Humanin administration has shown vascular and cardioprotective effects in preclinical models [24,25,26]. In our cohort, serum Humanin concentrations were higher in patients with hvKP-associated bloodstream infection and liver abscess than in matched healthy controls. Because serum samples were collected after initiation of antimicrobial therapy and at variable time points during the clinical course, potential effects of treatment and sampling timing on circulating Humanin concentrations cannot be excluded. In addition, the single-time-point measurement does not allow us to determine whether this increase was specifically related to hvKP infection, reflected disease severity, or represented a stress-associated or compensatory response. Its cellular source, temporal profile, and functional significance remain unknown. The relationship between serum Humanin concentrations and biochemical markers of liver injury or clinical disease severity was not evaluated in the present cohort and should be investigated in larger clinical studies.

Macrophages play context-dependent roles in *K. pneumoniae* infection. Although they contribute to bacterial recognition and clearance, excessive macrophage-associated inflammatory responses may also promote hepatic immunopathology during invasive hvKP infection [13,23]. Systemic hvKP infection increased hepatic F4/80 immunoreactivity and CD86 fluorescence but did not alter CD206 fluorescence. Because F4/80 and CD86 were measured separately, the data do not demonstrate M1 macrophage polarization. They instead indicate a stronger macrophage-associated pro-inflammatory response, which Humanin attenuated without significantly changing CD206 expression. In parallel, liver transcriptomics showed lower expression of multiple pro-inflammatory genes and negative enrichment of NF-κB signaling after Humanin pretreatment, whereas Western blotting showed reduced p-p65/p65 and p-IκBα/IκBα ratios in liver tissue and hvKP-stimulated BMDMs, particularly at a higher dose or concentration. These complementary findings are consistent with reduced NF-κB activation after Humanin pretreatment but do not establish that NF-κB signaling mediates the protective effect of Humanin. Studies using macrophage-specific NF-κB manipulation are required in the future to establish the causal role of this signaling axis.

Previous studies and reviews have described the anti-inflammatory properties of Humanin and its derivatives in retinal endothelial injury and neuroinflammatory conditions [27,28]. Our results extend these observations to systemic bacterial infection. Humanin attenuated inflammation in mouse liver tissue and hvKP-stimulated BMDMs, with the most consistent effects at 5 mg/kg in vivo and 50 μM in vitro. The 25 and 50 μM concentrations used in BMDMs were substantially higher than the endogenous circulating Humanin concentrations observed in our clinical cohort; therefore, they should be regarded as pharmacological in vitro exposures rather than physiological equivalents of serum Humanin levels. Reduced NF-κB activation accompanied these effects, and the higher in vivo dose prolonged survival. These findings support the need for a further investigation into the protective effects of Humanin during hvKP infection. Because inflammation also contributes to antibacterial defense, future experiments should determine whether Humanin remains protective when administered after infection or with antimicrobial therapy. To the best of our knowledge, this is the first report demonstrating a protective effect of Humanin pretreatment in an experimental model of systemic hvKP infection. However, the specific molecular targets through which Humanin exerts its effects remain to be further explored.

This study has several limitations that must be acknowledged. Firstly, while Humanin pretreatment was associated with reduced NF-κB activation and inflammatory factor levels in both liver tissue and BMDMs, the direct molecular targets through which Humanin exerts its effects remain unclear. Secondly, the Humanin pretreatment regimen employed in this experiment involved administering the peptide prior to mouse infection; however, in clinical settings, patients often present at various stages of infection. Therefore, it remains uncertain whether Humanin retains protective effects when administered after infection. In future studies, post-infection administration would more closely mimic the clinical timing of treatment and help determine whether Humanin retains its protective effects once infection is established, thereby strengthening the translational relevance of these findings. Thirdly, all infection experiments were performed using a single clinical hvKP isolate; therefore, strain-specific effects cannot be excluded, and the findings may not be generalizable to other hvKP strains. In addition, no formal a priori power calculation was performed, and the relatively small sample sizes may have limited the statistical power and precision of some of the comparisons. The histopathological assessment was qualitative, and no standardized quantitative scoring system was applied, which limits the precision of the comparisons of histological injury among groups. Finally, only male mice were used in the present study; therefore, the findings may not be generalizable to female mice.

## 5. Conclusions

In conclusion, Humanin pretreatment attenuated inflammatory liver injury during systemic hvKP infection, and 5 mg/kg prolonged survival in mice. The protective phenotype was associated with reduced macrophage-associated inflammation and NF-κB activation. These findings support the need for a further investigation into Humanin, including post-infection administration, while its molecular targets remain to be established.

## Figures and Tables

**Table 1:** Primers for quantitative real-time polymerase chain reaction (qRT-PCR) used in this study.

Gene		Primer Sequence (5′–3′)	Length of Product
*Tnf*	F	CTGAACTTCGGGGTGATCGG	122 bp
	R	GGCTTGTCACTCGAATTTTGAGA	
*Il* *6*	F	TCTATACCACTTCACAAGTCGGA	88 bp
	R	GAATTGCCATTGCACAACTCTTT	
*Il1b*	F	TGCCACCTTTTGACAGTGATG	138 bp
	R	TGATGTGCTGCTGCGAGATTT	
*Ccl2*	F	TAAAAACCTGGATCGGAACCAAA	120 bp
	R	GCATTAGCTTCAGATTTACGGGT	
*Gapdh*	F	AGGTCGGTGTGAACGGATTTG	123 bp
	R	TGTAGACCATGTAGTTGAGGTCA	

## Data Availability

The authors declare that the relevant data generated in this study are included in the article, and additional supporting data are available from the corresponding author upon reasonable request.

## Supplementary Materials

The following supporting information can be downloaded at https://www.mdpi.com/article/10.3390/pathogens15090966/s1, Figure S1: Bacterial burdens in mouse blood and liver following hvKP infection with or without Humanin pretreatment. Figure S2. Hepatic CD206 immunofluorescence does not differ significantly among the experimental groups. Figure S3. Principal component analysis of liver RNA-seq samples. Figure S4. Quantification of total p65 and IκBα protein expression in mouse liver tissue and BMDMs. Table S1. Clinical characteristics of patients with hvKP-associated bloodstream infection and liver abscess. Table S2. Overall statistical tests, effect estimates, 95% confidence intervals, and pairwise comparisons.

## Abbreviations

The following abbreviations are used in this manuscript:

hvKP	Hypervirulent *Klebsiella pneumoniae*
BSI	bloodstream infection
HN	Humanin
MPO	myeloperoxidase
BMDM	bone marrow-derived macrophage
DEG	Differentially expressed gene
PCT	Procalcitonin
ELISA	enzyme-linked immunosorbent assay
OD	optical density
LB	Luria–Bertani
ALT	alanine aminotransferase
AST	aspartate aminotransferase
DMEM	Dulbecco’s Modified Eagle Medium
ECL	enhanced chemiluminescence
GO	Gene Ontology
KEGG	Kyoto Encyclopedia of Genes and Genomes
BP	biological process
CC	cellular component
MF	molecular function
NF-κB	nuclear factor kappa B
CFU	colony-forming unit
PBS	phosphate-buffered saline
DPBS	Dulbecco’s phosphate-buffered saline
HRP	horseradish peroxidase
TMB	3,3′,5,5′-tetramethylbenzidine
MHA	Mueller–Hinton agar
DAB	3,3′-diaminobenzidine
DAPI	4′,6-diamidino-2-phenylindole
RIPA	radioimmunoprecipitation assay
BCA	bicinchoninic acid
GAPDH	glyceraldehyde-3-phosphate dehydrogenase
GSEA	gene-set enrichment analysis
NES	normalized enrichment score
FDR	false discovery rate
qRT-PCR	quantitative real-time polymerase chain reaction
