# Supplementary material for: Humanin Attenuates Inflammatory Liver Injury and NF-κB Activation During Systemic Hypervirulent Klebsiella pneumoniae Infection
Source: Pathogens. 2026 Sep 10;15(9):966. doi: 10.3390/pathogens15090966 (PMC13610375; doi:10.3390/pathogens15090966)
Supplement: Supplementary file 1 [file pathogens-15-00966-s001.zip › Supplementary Figures.pdf]

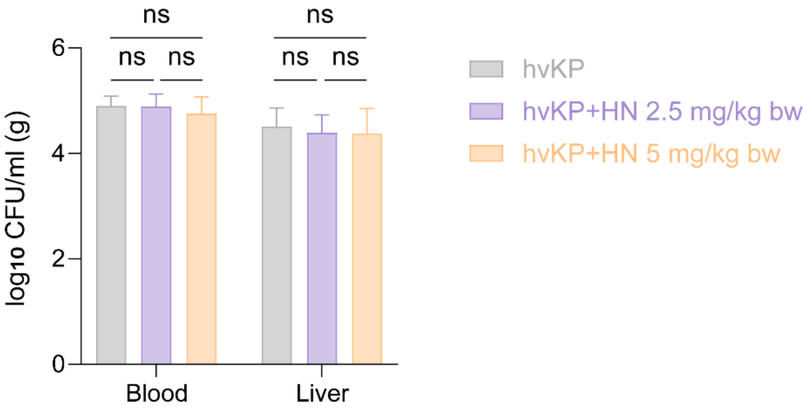

Figure S1. Bacterial burdens in mouse blood and liver following hvKP infection with or without Humanin pretreatment. Blood and liver bacterial burdens are expressed as $\log_{10}$ CFU/mL and $\log_{10}$ CFU/g, respectively. Data are presented as the mean ± SD (n = 5 per group). Statistical comparisons were performed using one-way ANOVA followed by Tukey's multiple-comparisons test. No statistically significant differences in blood or hepatic bacterial burden were detected between the Humanin-pretreated and untreated hvKP groups. ns, not significant. HN, Humanin; hvKP, hypervirulent *Klebsiella pneumoniae*; CFU, colony-forming units; bw, body weight.

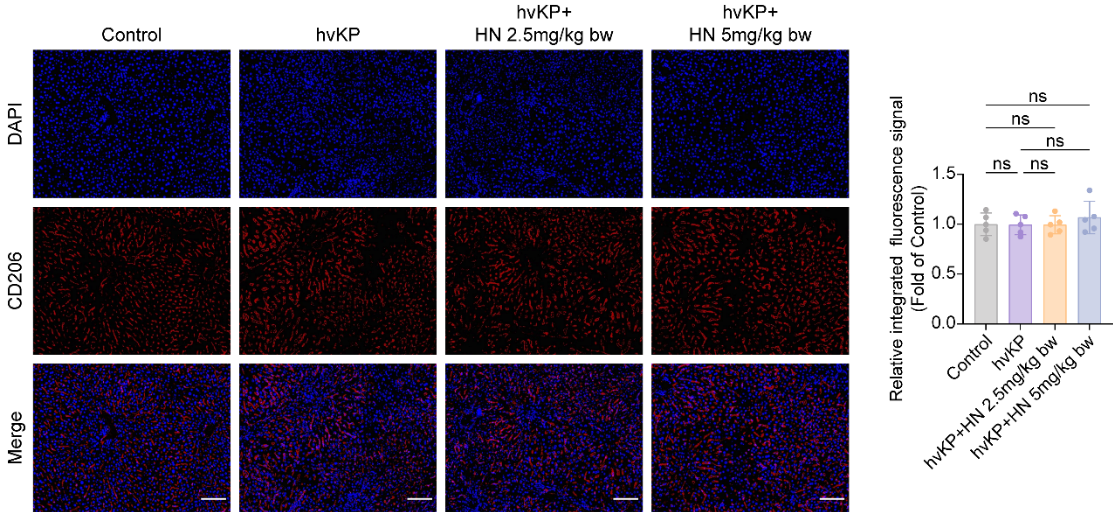

Figure S2. Hepatic CD206 immunofluorescence does not differ significantly among the experimental groups. Representative immunofluorescence images of CD206 expression in liver sections from uninfected control mice, untreated hvKP-infected mice, and hvKP-infected

mice pretreated with Humanin at 2.5 or 5 mg/kg. CD206 is shown in red, nuclei were counterstained with DAPI (blue), and merged images are shown in the lower panels. The graph shows the integrated CD206 fluorescence intensity relative to the uninfected control group. Each point represents one mouse, and bars show the mean ± SD (n = 5 per group). Statistical comparisons were performed using ordinary one-way ANOVA followed by Tukey's multiple-comparisons test. No statistically significant differences were detected among the groups. Scale bars, 100 μm; ns, not significant; HN, Humanin; hvKP, hypervirulent *Klebsiella pneumoniae*; bw, body weight.

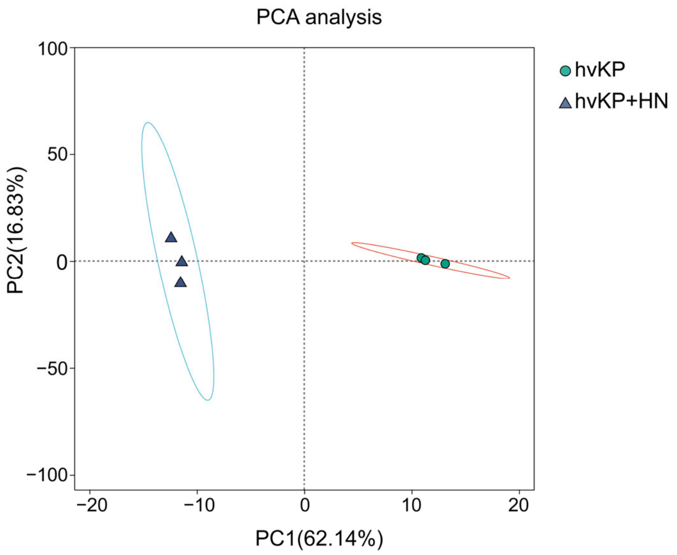

Figure S3. Principal component analysis of liver RNA-seq samples. PCA of transcriptomic profiles from the hvKP and hvKP + Humanin groups (n = 3 biological replicates per group). PC1 and PC2 explained 62.14% and 16.83% of the total variance, respectively. Samples separated according to experimental group. HN, Humanin; hvKP, hypervirulent *Klebsiella pneumoniae*.

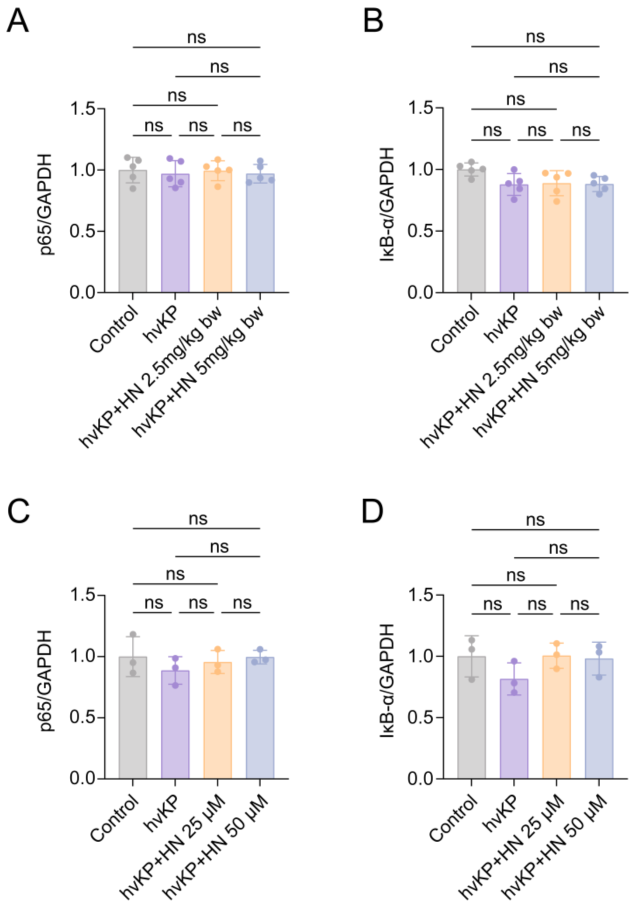

Figure S4. Quantification of total p65 and IκBα protein expression in mouse liver tissue and BMDMs. (A, B) Densitometric quantification of total p65/GAPDH (A) and IκBα/GAPDH (B) ratios in mouse liver tissue. Each point represents one mouse, and bars show the mean ± SD (n = 5 per group). (C, D) Densitometric quantification of total p65/GAPDH (C) and IκBα/GAPDH (D) ratios in BMDMs. Each point represents one independent biological replicate, and bars show the mean ± SD (n = 3 per group). Statistical comparisons were performed using ordinary one-way ANOVA followed by Tukey's multiple-comparisons test. No statistically significant differences were detected among the corresponding experimental groups. ns, not significant; HN, Humanin; hvKP, hypervirulent *Klebsiella pneumoniae*.
