# Supplementary material for: Humanin Attenuates Inflammatory Liver Injury and NF-κB Activation During Systemic Hypervirulent Klebsiella pneumoniae Infection
Source: Pathogens. 2026 Sep 10;15(9):966. doi: 10.3390/pathogens15090966 (PMC13610375; doi:10.3390/pathogens15090966)
Supplement: Supplementary file 1 [file pathogens-15-00966-s001.zip › Supplementary Table S1.pdf]

Table S1. Clinical characteristics of patients with hvKP-associated bloodstream infection and liver abscess.

Patient No.	ICU admission	Additional disease severity indicators	Major comorbidities	Organ dysfunction	Antimicrobial treatment	Serum sampling time after diagnosis and initiation of antimicrobial therapy
P01	No	Not available	Diabetes mellitus; hypertension	None	Meropenem; piperacillin/tazobactam	72 h
P02	Yes	Septic shock	Decompensated hepatitis B virus-related cirrhosis; coronary atherosclerosis; arrhythmia	Renal insufficiency; cardiac insufficiency	Meropenem; piperacillin/sulbactam	5 days
P03	Yes	APACHE II 9; SOFA 6	Diabetes mellitus; fatty liver disease; cholelithiasis	Hepatic dysfunction	Meropenem; cefoperazone/sulbactam	5 days
P04	Yes	Septic shock; APACHE II 14; SOFA 8	None	Hepatic dysfunction	Meropenem	24 h
P05	Yes	Septic shock	Diabetes mellitus	Hepatic dysfunction	Meropenem	48 h
P06	Yes	APACHE II 11; SOFA 8; GCS 12	Alzheimer's disease; chronic cholecystitis; aortic atherosclerosis; chronic pneumonia	Hepatic dysfunction	Meropenem	48 h
P07	Yes	SOFA 5; mechanical ventilation	Diabetes mellitus; hypertension; nephrolithiasis	Multiple-organ failure (respiratory, hepatic, renal, and coagulation); type I respiratory failure	Meropenem	72 h
P08	Yes	APACHE II 23; mechanical ventilation	Diabetes mellitus	Type I respiratory failure; renal insufficiency	Meropenem	72 h
P09	Yes	Septic shock; APACHE II 17; SOFA 5; GCS 15; mechanical ventilation	Diabetes mellitus; chronic pneumonia	Hepatic dysfunction; circulatory failure; renal insufficiency	Meropenem; cefoperazone/sulbactam	72 h
P10	Yes	SOFA 5	Hypertension; cholelithiasis; aortic atherosclerosis	Hepatic dysfunction	Meropenem; cefoperazone/sulbactam	72 h
P11	No	Not available	Chronic pneumonia	Hepatic dysfunction	Cefoperazone/sulbactam	5 days
P12	No	Not available	Chronic hepatitis B; hypertriglyceridemia; fatty liver disease	None	Cefoperazone/sulbactam	24 h
P13	No	Not available	Hepatitis B surface antigen (HBsAg) carrier; fatty liver disease; benign prostatic hyperplasia	Not available	Cefoperazone/sulbactam	5 days
P14	No	Not available	Coronary and aortic atherosclerosis; gout;	Hepatic dysfunction	Ceftriaxone	5 days

			arthritis; atrophic gastritis			
P15	No	Not available	Fatty liver disease	None	Meropenem; cefoperazone/sulbactam	5 days
P16	No	Not available	Diabetes mellitus; hypertension; hyperlipidemia; chronic cholecystitis; benign prostatic hyperplasia	Hepatic dysfunction	Ertapenem	24 h
P17	No	Not available	Hypertension; nephrolithiasis	None	Cefoperazone/sulbactam	24 h
P18	No	Not available	Fatty liver disease	None	Cefoperazone/sulbactam	72 h
P19	No	Not available	Diabetes mellitus; chronic hepatitis B; aortic atherosclerosis	Hepatic dysfunction	Meropenem	24 h
P20	No	Not available	Fatty liver disease	Hepatic dysfunction	Cefoperazone/sulbactam	5 days

Note: All patients were diagnosed with sepsis. Antimicrobial therapy was initiated at the time of clinical diagnosis in all patients; serum samples were collected 24 h to 5 days thereafter. Sepsis is not repeated in the "Additional disease severity indicators" column. APACHE II, SOFA, and GCS scores are reported when available. Comorbidities were summarized from diagnoses documented in the available clinical records. "None" indicates that no corresponding major comorbidity or organ dysfunction was identified in the available clinical records. "Not available" indicates that the corresponding information was not available in the clinical records.

Abbreviations: APACHE II, Acute Physiology and Chronic Health Evaluation II; GCS, Glasgow Coma Scale; HBsAg, hepatitis B surface antigen; ICU, intensive care unit; SOFA, Sequential Organ Failure Assessment.
