# Supplementary material for: Humanin Attenuates Inflammatory Liver Injury and NF-κB Activation During Systemic Hypervirulent Klebsiella pneumoniae Infection
Source: Pathogens. 2026 Sep 10;15(9):966. doi: 10.3390/pathogens15090966 (PMC13610375; doi:10.3390/pathogens15090966)
Supplement: Supplementary file 1 [file pathogens-15-00966-s001.zip › Supplementary Table S2.pdf]

Table S2. Overall statistical tests, effect estimates, 95% confidence intervals, and pairwise comparisons.

Figure	Outcome	Overall statistical test	Overall effect size	Comparison	Effect estimate / mean difference	95% CI	P value
**Figure 1**	**Serum Humanin concentration (pg/mL)**	Two-tailed Mann–Whitney U test	—	hvKP patients vs Healthy controls	Hodges–Lehmann location shift = 388.9 pg/mL	202.4 to 686.9	<0.001
**Figure 2C**	**ALT**	Ordinary one-way ANOVA: F(3,16) = 40.64, P < 0.0001	η² = 0.884	Control vs hvKP	−2695	−3451 to −1940	<0.0001
				Control vs hvKP + HN 2.5 mg/kg	−2309	−3064 to −1554	<0.0001
				Control vs hvKP + HN 5 mg/kg	−1619	−2375 to −863.9	<0.0001
				hvKP vs hvKP + HN 2.5 mg/kg	386.4	−369.1 to 1142	0.4809
				hvKP vs hvKP + HN 5 mg/kg	1076	320.5 to 1831	0.0044
				hvKP + HN 2.5 mg/kg vs hvKP + HN 5 mg/kg	689.6	−65.88 to 1445	0.0800
**Figure 2D**	**AST**	Ordinary one-way ANOVA: F(3,16) = 27.71, P < 0.0001	η² = 0.839	Control vs hvKP	−2964	−3962 to −1966	<0.0001
				Control vs hvKP + HN 2.5 mg/kg	−2427	−3425 to −1429	<0.0001
				Control vs hvKP + HN 5 mg/kg	−1496	−2494 to −498.4	0.0028
				hvKP vs hvKP + HN 2.5 mg/kg	536.8	−461.0 to 1535	0.4387
				hvKP vs hvKP + HN 5 mg/kg	1468	470.0 to 2466	0.0034
				hvKP + HN 2.5 mg/kg vs hvKP + HN 5 mg/kg	931.0	−66.83 to 1929	0.0718
**Figure 2E**	**PCT**	Welch’s one-way ANOVA: W(3.000,6.700) = 137.5, P < 0.0001	—	Control vs hvKP	−3804	−4615 to −2993	0.0001
				Control vs hvKP + HN 2.5 mg/kg	−3179	−5022 to −1336	0.0072
				Control vs hvKP + HN 5 mg/kg	−2301	−4578 to −24.15	0.0483
				hvKP vs hvKP + HN 2.5 mg/kg	625.1	−1184 to 2434	0.6963
				hvKP vs hvKP + HN 5 mg/kg	1503	−669.3 to 3676	0.1744
				hvKP + HN 2.5 mg/kg vs hvKP + HN 5 mg/kg	878.3	−1378 to 3135	0.7196
**Figure 2F**	**Hepatic MPO activity**	Ordinary one-way ANOVA: F(3,16) = 15.73, P < 0.0001	η² = 0.747	Control vs hvKP	−1.039	−1.489 to −0.5891	<0.0001
				Control vs hvKP + HN 2.5	−0.7641	−1.214 to −0.3141	0.0009

				mg/kg			
				Control vs hvKP + HN 5 mg/kg	−0.5285	−0.9785 to −0.07843	0.0188
				hvKP vs hvKP + HN 2.5 mg/kg	0.2750	−0.1750 to 0.7251	0.3326
				hvKP vs hvKP + HN 5 mg/kg	0.5107	0.06062 to 0.9607	0.0235
				hvKP + HN 2.5 mg/kg vs hvKP + HN 5 mg/kg	0.2356	−0.2144 to 0.6857	0.4615
**Figure S1**	**Blood bacterial burden (log10 CFU/mL)**	Ordinary one-way ANOVA: F(2,12) = 0.5043, P = 0.6162	$\eta^2 = 0.0775$	hvKP vs hvKP + HN 2.5 mg/kg	0.009293	−0.4170 to 0.4356	0.9981
				hvKP vs hvKP + HN 5 mg/kg	0.1434	−0.2829 to 0.5697	0.6521
				hvKP + HN 2.5 mg/kg vs hvKP + HN 5 mg/kg	0.1341	−0.2922 to 0.5604	0.6869
**Figure S1**	**Liver bacterial burden (log10 CFU/g)**	Ordinary one-way ANOVA: F(2,12) = 0.1693, P = 0.8462	$\eta^2 = 0.0274$	hvKP vs hvKP + HN 2.5 mg/kg	0.1174	−0.5414 to 0.7763	0.8840
				hvKP vs hvKP + HN 5 mg/kg	0.1304	−0.5284 to 0.7893	0.8592
				hvKP + HN 2.5 mg/kg vs hvKP + HN 5 mg/kg	0.01298	−0.6459 to 0.6718	0.9985
**Figure 3A**	**F4/80-positive area (%)**	Ordinary one-way ANOVA: F(3,16) = 61.61, P < 0.0001	$\eta^2 = 0.920$	Control vs hvKP	−11.00	−13.44 to −8.551	<0.0001
				Control vs hvKP + HN 2.5 mg/kg	−8.740	−11.19 to −6.294	<0.0001
				Control vs hvKP + HN 5 mg/kg	−6.290	−8.735 to −3.844	<0.0001
				hvKP vs hvKP + HN 2.5 mg/kg	2.257	−0.1886 to 4.703	0.0758
				hvKP vs hvKP + HN 5 mg/kg	4.707	2.261 to 7.153	0.0003
				hvKP + HN 2.5 mg/kg vs hvKP + HN 5 mg/kg	2.450	0.004339 to 4.896	0.0495
**Figure 3B**	**Relative integrated CD86 fluorescence intensity**	Ordinary one-way ANOVA: F(3,16) = 43.09, P < 0.0001	$\eta^2 = 0.890$	Control vs hvKP	−74.34	−94.60 to −54.09	<0.0001
				Control vs hvKP + HN 2.5 mg/kg	−51.41	−71.67 to −31.16	<0.0001
				Control vs hvKP + HN 5 mg/kg	−20.60	−40.86 to −0.3444	0.0455
				hvKP vs hvKP + HN 2.5 mg/kg	22.93	2.675 to 43.19	0.0239
				hvKP vs hvKP + HN 5 mg/kg	53.74	33.49 to 74.00	<0.0001
				hvKP + HN 2.5 mg/kg vs hvKP + HN 5 mg/kg	30.81	10.56 to 51.07	0.0025
**Figure S2**	**CD206 fluorescence intensity**	Ordinary one-way ANOVA: F(3,16) = 0.4495, P	$\eta^2 = 0.0777$	Control vs hvKP	0.005790	−0.2117 to 0.2233	0.9998

		= 0.7211					
				Control vs hvKP + HN 2.5 mg/kg	0.005463	−0.2120 to 0.2229	0.9999
				Control vs hvKP + HN 5 mg/kg	−0.06812	−0.2856 to 0.1493	0.8069
				hvKP vs hvKP + HN 2.5 mg/kg	−0.0003268	−0.2178 to 0.2171	>0.9999
				hvKP vs hvKP + HN 5 mg/kg	−0.07391	−0.2914 to 0.1436	0.7667
				hvKP + HN 2.5 mg/kg vs hvKP + HN 5 mg/kg	−0.07359	−0.2911 to 0.1439	0.7691
**Figure 4A**	**Tnf mRNA expression**	Ordinary one-way ANOVA: $F(3,16) = 21.19$, $P < 0.0001$	$\eta^2 = 0.799$	Control vs hvKP	−98.22	−135.1 to −61.29	<0.0001
				Control vs hvKP + HN 2.5 mg/kg	−74.89	−111.8 to −37.97	0.0001
				Control vs hvKP + HN 5 mg/kg	−51.60	−88.53 to −14.68	0.0051
				hvKP vs hvKP + HN 2.5 mg/kg	23.32	−13.60 to 60.25	0.3059
				hvKP vs hvKP + HN 5 mg/kg	46.61	9.689 to 83.54	0.0113
				hvKP + HN 2.5 mg/kg vs hvKP + HN 5 mg/kg	23.29	−13.64 to 60.21	0.3072
**Figure 4B**	**Il6 mRNA expression**	Ordinary one-way ANOVA: $F(3,16) = 21.44$, $P < 0.0001$	$\eta^2 = 0.801$	Control vs hvKP	−170.5	−234.7 to −106.4	<0.0001
				Control vs hvKP + HN 2.5 mg/kg	−86.99	−151.1 to −22.84	0.0065
				Control vs hvKP + HN 5 mg/kg	−39.29	−103.4 to 24.86	0.3308
				hvKP vs hvKP + HN 2.5 mg/kg	83.55	19.39 to 147.7	0.0089
				hvKP vs hvKP + HN 5 mg/kg	131.2	67.09 to 195.4	0.0001
				hvKP + HN 2.5 mg/kg vs hvKP + HN 5 mg/kg	47.70	−16.46 to 111.9	0.1867
**Figure 4C**	**Il1b mRNA expression**	Ordinary one-way ANOVA: $F(3,16) = 29.78$, $P < 0.0001$	$\eta^2 = 0.848$	Control vs hvKP	−119.1	−158.1 to −80.03	<0.0001
				Control vs hvKP + HN 2.5 mg/kg	−68.17	−107.2 to −29.12	0.0007
				Control vs hvKP + HN 5 mg/kg	−22.73	−61.78 to 16.32	0.3729
				hvKP vs hvKP + HN 2.5 mg/kg	50.91	11.86 to 89.96	0.0089
				hvKP vs hvKP + HN 5 mg/kg	96.35	57.30 to 135.4	<0.0001
				hvKP + HN 2.5 mg/kg vs hvKP + HN 5 mg/kg	45.44	6.390 to 84.49	0.0199
**Figure 4D**	**Ccl2 mRNA expression**	Welch’s one-way ANOVA:	—	Control vs hvKP	−501.7	−700.2 to −303.1	0.0017

		W(3.000,6.669) = 211.6, P < 0.0001					
				Control vs hvKP + HN 2.5 mg/kg	−297.9	−351.7 to −244.1	<0.0001
				Control vs hvKP + HN 5 mg/kg	−136.2	−215.7 to −56.71	0.0074
				hvKP vs hvKP + HN 2.5 mg/kg	203.8	18.70 to 388.8	0.0344
				hvKP vs hvKP + HN 5 mg/kg	365.5	173.1 to 557.9	0.0033
				hvKP + HN 2.5 mg/kg vs hvKP + HN 5 mg/kg	161.7	84.99 to 238.4	0.0008
**Figure 6B**	**Liver p-p65/p65 ratio**	Ordinary one-way ANOVA: $F(3,16) = 33.59$, $P < 0.0001$	$\eta^2 = 0.863$	Control vs hvKP	−1.022	−1.383 to −0.6601	<0.0001
				Control vs hvKP + HN 2.5 mg/kg	−0.4821	−0.8435 to −0.1207	0.0074
				Control vs hvKP + HN 5 mg/kg	0.1116	−0.2498 to 0.4730	0.8133
				hvKP vs hvKP + HN 2.5 mg/kg	0.5394	0.1780 to 0.9008	0.0030
				hvKP vs hvKP + HN 5 mg/kg	1.133	0.7717 to 1.495	<0.0001
				hvKP + HN 2.5 mg/kg vs hvKP + HN 5 mg/kg	0.5937	0.2324 to 0.9551	0.0012
**Figure 6C**	**Liver p-IκBα/IκBα ratio**	Ordinary one-way ANOVA: $F(3,16) = 10.95$, $P = 0.0004$	$\eta^2 = 0.672$	Control vs hvKP	−0.7675	−1.251 to −0.2843	0.0017
				Control vs hvKP + HN 2.5 mg/kg	−0.6041	−1.087 to −0.1209	0.0121
				Control vs hvKP + HN 5 mg/kg	−0.02289	−0.5061 to 0.4603	0.9991
				hvKP vs hvKP + HN 2.5 mg/kg	0.1634	−0.3198 to 0.6467	0.7693
				hvKP vs hvKP + HN 5 mg/kg	0.7446	0.2614 to 1.228	0.0022
				hvKP + HN 2.5 mg/kg vs hvKP + HN 5 mg/kg	0.5812	0.09798 to 1.064	0.0159
**Figure 6E**	**BMDM p-p65/p65 ratio**	Ordinary one-way ANOVA: $F(3,8) = 9.315$, $P = 0.0055$	$\eta^2 = 0.777$	Control vs hvKP	−0.6819	−1.173 to −0.1907	0.0093
				Control vs hvKP + HN 25 μM	−0.3210	−0.8122 to 0.1702	0.2338
				Control vs hvKP + HN 50 μM	0.02338	−0.4678 to 0.5146	0.9986
				hvKP vs hvKP + HN 25 μM	0.3609	−0.1303 to 0.8521	0.1647
				hvKP vs hvKP + HN 50 μM	0.7053	0.2141 to 1.196	0.0076
				hvKP + HN 25 μM vs hvKP + HN 50 μM	0.3444	−0.1468 to 0.8356	0.1907
**Figure 6F**	**BMDM p-IκBα/IκBα ratio**	Ordinary one-way ANOVA: $F(3,8) = 12.90$, $P =$	$\eta^2 = 0.829$	Control vs hvKP	−1.091	−1.701 to −0.4802	0.0020

		0.0020					
				Control vs hvKP + HN 25 μM	−0.5844	−1.195 to 0.02609	0.0606
				Control vs hvKP + HN 50 μM	−0.1796	−0.7900 to 0.4309	0.7842
				hvKP vs hvKP + HN 25 μM	0.5063	−0.1042 to 1.117	0.1081
				hvKP vs hvKP + HN 50 μM	0.9110	0.3006 to 1.521	0.0061
				hvKP + HN 25 μM vs hvKP + HN 50 μM	0.4048	−0.2057 to 1.015	0.2246
**Figure S4A**	**Liver total p65/GAPDH**	Ordinary one-way ANOVA: $F(3,16) = 0.1467$, $P = 0.9303$	$\eta^2 = 0.02677$	Control vs hvKP	0.03014	−0.1383 to 0.1986	0.9551
				Control vs hvKP + HN 2.5 mg/kg	0.005235	−0.1632 to 0.1737	0.9997
				Control vs hvKP + HN 5 mg/kg	0.02983	−0.1386 to 0.1983	0.9563
				hvKP vs hvKP + HN 2.5 mg/kg	−0.02490	−0.1934 to 0.1436	0.9737
				hvKP vs hvKP + HN 5 mg/kg	−0.0003076	−0.1688 to 0.1682	>0.9999
				hvKP + HN 2.5 mg/kg vs hvKP + HN 5 mg/kg	0.02460	−0.1439 to 0.1931	0.9746
**Figure S4B**	**Liver total IκBα/GAPDH**	Ordinary one-way ANOVA: $F(3,16) = 2.790$, $P = 0.0741$	$\eta^2 = 0.3435$	Control vs hvKP	0.1223	−0.02018 to 0.2649	0.1061
				Control vs hvKP + HN 2.5 mg/kg	0.1116	−0.03090 to 0.2541	0.1545
				Control vs hvKP + HN 5 mg/kg	0.1181	−0.02443 to 0.2606	0.1234
				hvKP vs hvKP + HN 2.5 mg/kg	−0.01073	−0.1532 to 0.1318	0.9963
				hvKP vs hvKP + HN 5 mg/kg	−0.004251	−0.1468 to 0.1383	0.9998
				hvKP + HN 2.5 mg/kg vs hvKP + HN 5 mg/kg	0.006474	−0.1360 to 0.1490	0.9992
**Figure S4C**	**BMDM total p65/GAPDH**	Ordinary one-way ANOVA: $F(3,8) = 0.6504$, $P = 0.6046$	$\eta^2 = 0.1961$	Control vs hvKP	0.1135	−0.1826 to 0.4095	0.6283
				Control vs hvKP + HN 25 μM	0.04397	−0.2520 to 0.3400	0.9624
				Control vs hvKP + HN 50 μM	0.003596	−0.2924 to 0.2996	>0.9999
				hvKP vs hvKP + HN 25 μM	−0.06948	−0.3655 to 0.2265	0.8735
				hvKP vs hvKP + HN 50 μM	−0.1099	−0.4059 to 0.1862	0.6501
				hvKP + HN 25 μM vs hvKP + HN 50 μM	−0.04037	−0.3364 to 0.2556	0.9704
**Figure S4D**	**BMDM total IκBα/GAPDH**	Ordinary one-way ANOVA: $F(3,8) = 1.328$, $P = 0.3314$	$\eta^2 = 0.3325$	Control vs hvKP	0.1845	−0.1714 to 0.5405	0.4014
				Control vs hvKP + HN 25 μM	−0.005014	−0.3610 to 0.3509	>0.9999
				Control vs hvKP + HN 50 μM	0.01846	−0.3375 to 0.3744	0.9982

	hvKP vs hvKP + HN 25 μM	−0.1895	−0.5455 to 0.1664	0.3807
	hvKP vs hvKP + HN 50 μM	−0.1661	−0.5220 to 0.1899	0.4832
	hvKP + HN 25 μM vs hvKP + HN 50 μM	0.02347	−0.3325 to 0.3794	0.9964

Note: For ordinary one-way ANOVA, overall effect size is reported as eta squared (η^2). Tukey-adjusted pairwise mean differences and 95% confidence intervals are shown for ordinary ANOVA outcomes. For heteroscedastic outcomes analyzed by Welch's one-way ANOVA, Dunnett's T3-adjusted pairwise comparisons are shown. For Figure 1, the effect estimate is the Hodges–Lehmann location shift with 95% confidence interval. Mean differences are calculated as the first group minus the second group.
